# Transcriptome analysis revealed the potential molecular mechanism of style bending movement in passion fruit (*Passiflora Edulis* Sims)

**DOI:** 10.1186/s12870-025-07431-8

**Published:** 2025-10-29

**Authors:** Ling Gao, Jie Wang, Li Xu, Yanan Zhao, Siying Wu, Mei Chen, Dongshan Ying, Linhua Wu, Fusun Yang, Guodao Liu

**Affiliations:** 1 College of Plant Science and Technology of Huazhong Agricultural University, Wuhan , 430070 China; 2 Tropical Crops Genetic Resources Institute, Chinese Academy of Tropical Agricultural Sciences. Key Laboratory of Crop Gene Resources and Germplasm Enhancement in Southern China, Ministry of Agriculture and Rual Affairs. Key Laboratory of Tropical Crops Germplasm Resources Genetic Improvement and Innovation of Hainan Province, Haikou, 571101 China; 3 Sanya Research Institute, Chinese Academy of Tropical Agricultural Science, Sanya, 572024 China; 4 Institute of Tropical Agriculture and Forestry, Hainan University, 571737 Haikou, China

**Keywords:** Heat shock proteins, Lignin biosynthesis, Passion fruit, Phytohormones, Style bending movement, Transcriptome analysis

## Abstract

Passion fruit has a difficult natural fruiting process and low yield due to its approach herkogamy. Understanding the molecular mechanism of passion fruit style bending is crucial as it enhances pollination efficiency by optimizing stigma-anther alignment, overcoming herkogamy and improving fruit yield. In this study, we used the purple passion fruit variety ‘Tainong 1’ as experimental material. We observed that its style bends into an “S”-shape after flowering. As development progresses, the degree of this S-shaped curvature gradually weakens. Simultaneously, the style also exhibits a spatial bending from top to bottom during this process. In order to identify the genes specifically induced during the style bending process, transcriptome analysis was conducted on styles at T_1_ (flowering), T_2_ (30 min post-flowering), and T_5_ (120 min post-flowering). 226 DEGs were identified to be specifically expressed in the T_1_ to T_2_ stages, mainly enriched in the “Protein processing in endoplasmic reticulum” pathway, involving heat shock proteins and DnaJ family proteins, suggesting that heat stress response is involved in early style movement regulation. The enriched 18 DEGs were all heat shock protein family proteins and DnaJ family proteins, which may be due to the stress response of the style to environmental heat stimulation. At the same time, 1,080 DEGs were specifically expressed in the T_2_ to T_5_ stages, mainly enriched in the “Phenylpropanoid biosynthesis” pathway and involved in the lignin biosynthesis pathway. Physiological testing confirmed that as the S-shaped of the style weakens, lignin accumulates significantly, increasing by 60% from T_1_ to T_2_ and by 43% from T_2_ to T_5_, which is consistent with the trend of transcriptome data. In addition, differential expression of hormone metabolism related genes (abscisic acid, brassino steroids, jasmonic acid, and gibberellin pathways) was observed. Endogenous hormone quantification further confirmed hormonal regulation of style bending, revealing distinct accumulation patterns: ABA showed a biphasic trend with an initial 30% increase (T_1_ to T_2_) followed by a 9% rise (T_2_ to T_5_), while jasmonic acid exhibited a sharper 30% (T_1_ to T_2_) to 22% (T_2_ to T_5_) escalation. In summary, our results indicate that lignin accumulation enhances the mechanical strength of the style and reduces its inherent S-shaped bending. Thermal stimulation and hormone synthesis metabolism may also play a role in the process of style bending, but further research is needed to determine the specific mechanisms by which the response is regulated.

**Supplementary Information:**

The online version contains supplementary material available at 10.1186/s12870-025-07431-8.

## Introduction

Enhancing passion fruit (*Passiflora edulis* Sims.) yield has emerged as a pivotal research focus in tropical crop science [1, 2]. Known as the “king of juices,” this economically valuable crop shows significant potential for development in tropical and subtropical regions [3]. According to FAO statistics, global passion fruit production exceeded 1.2 million tons in 2024, with China accounting for 980,000 tons—representing 82% of the world’s total output. By 2025, global production is projected to surpass 5 million tons, while China’s cultivation area is expected to expand to over 220,000 hectares (approximately 1.47 million mu). Despite this significant production volume, China faces a striking supply-demand imbalance. Customs data from 2024 reveals an 85% import dependency for processed passion fruit products, with annual concentrated juice imports reaching 23,000 tons—98.5% of which were sourced from Vietnam. In stark contrast, China’s exports remained negligible at less than 1 ton. This severe disparity underscores the urgent need to improve yield and processing capabilities in passion fruit cultivation to bridge the gap between domestic production and market demand.

The unique herkogamy of passion fruit flowers, where the style extends beyond the anthers, significantly limits natural pollination efficiency. During flowering, the style undergoes a dynamic spatial movement: initially upright, it bends downward to position the stigma among the anthers [4]. This spatial repositioning critically influences pollen-pistil interaction [5–7]. Early research by Nishida first established the association between stigma movement and pollination success [8]. Notably, studies on sour passion fruit revealed that flowers with five carpels exhibit complete style curvature, achieving 100% pollination efficiency-a trait more prevalent in male parents [9, 10]. However, the molecular mechanisms driving style bending remain unknown. Additionally, recent studies demonstrate that cobalt nanoparticle (Co-NPs) treatment can significantly improve passion fruit micropropagation efficiency (with shoot induction rate increasing to 66.67%) by regulating the auxin/cytokinin ratio, suggesting the crucial role of endogenous hormone balance in organ morphogenesis [11]. Unraveling these mechanisms could provide key insights for improving passion fruit yield and fruit set rates.

‘Tainong 1’, a leading purple passion fruit variety extensively cultivated in regions like Fujian and Hainan, offers direct application value for research findings [12]. This cultivar exhibits stable agronomic traits, including vigorous growth, continuous flowering, uniform fruit size, and a single fruit weight of 80–120 g, facilitating reproducible experiments [13]. Its distinctive floral characteristics, notably prominent style curvature and a high proportion of pentacarpellate flowers (>30%), are advantageous for observing the relationship between pollination efficiency and morphogenesis [14]. Existing transcriptomic data can support the analysis of hormone signaling pathways [15]. Furthermore, ‘Tainong 1’ demonstrates excellent performance in pollination compatibility studies, exhibiting superior self-compatibility compared to other lines (TN >H2 >MB >H6). The fruit-setting rate and pollen tube growth behavior in cross-pollination combinations (e.g., H6 × TN) are highly representative, making this cultivar an ideal model for investigating the mechanisms underlying style movement [16].

The transcriptome represents the complete set of gene transcripts in an organism under specific conditions, and transcriptome analysis serves as a powerful tool for identifying genes associated with complex traits [17, 18]. Transcriptome-based studies have revealed floral organ development mechanisms in fruits like longan, dragon fruit, lychee, and pineapple, identifying numerous differentially expressed genes (DEGs) that regulate floral growth [19–22]. However, the genetic regulatory mechanism underlying passion fruit style bending remains unexplored. This study employed transcriptomic analysis of styles at distinct developmental stages during the flowering process of purple passion fruit to identify key regulatory genes governing style bending movement. The findings not only elucidate the molecular mechanisms underlying style curvature but also provide novel theoretical foundations for yield improvement in passion fruit cultivation.

## Materials and methods

### Plant materials and dynamic observation of style bending

The purple passion fruit variety ‘Tainong 1’ was used as the experimental material. It was planted on October 14, 2022 in a greenhouse (19.51° N, 109.51° E) of the Tropical Crop Variety Resources Research Institute of the Chinese Academy of Tropical Agricultural Sciences, with a spacing of 3 m between plants and 2 m between rows, totaling 40 plants. Cultivation management included: drip irrigation twice weekly (adjusted for soil moisture), monthly application of 15-15-15 NPK fertilizer at 200 kg/ha and integrated pest management combining neem oil (1% v/v, biweekly application) with Trichoderma-based biocontrol agents.

‘Tainong 1’ blooms from March to April every year. When the petals are fully open, the distance between the stigma and the anther is measured using a transparent ruler. From the total of 40 plants, we randomly sampled 30 plants and selected one flower per plant when the petals were fully opened (designated as T_1_). These 30 marked flowers were subsequently measured at 30-minute intervals.

### Slicing and microstructural observation

Manual slicing: Using a single-sided blade, cut the style into 0.1–0.2 mm sections by hand, and then observe under an SZN71 stereomicroscope (Shunyu Instrument Co., Ltd., Yuyao, China).

Paraffin sectioning: The styles (including stigma) were collected at three stages: fully open (T_1_), 30 min (T_2_), and 120 min (T_5_) after flowering. Styles were longitudinally bisected along the ventral sulcus (transverse stigma sections were cut perpendicular to the sulcus). Samples were fixed in FAA at 25 °C for 48 h, a duration optimized to preserve cytoarchitecture without over-fixation artifacts. Subsequent dehydration, embedding, sectioning (8 μm), and toluidine blue O staining (0.1% w/v, pH 4.5) followed He et al., for structural analysis [23]. Observe and scan paraffin sections using the Panoramic MIDI slicing digital scanner (3DHISTECH Ltd, Budapest, Hungary). Cell morphometric analysis was conducted using CaseViewer 2.4 software, with 10 epidermal cells randomly selected per section from three non-consecutive sections per flower (*n* = 5 flowers/stage). Length and width were measured along cellular longitudinal and transverse axes, respectively, with final values representing means of 150 measurements (30 cells/flower × 5 replicates) per developmental stage.

### RNA extraction and sequencing

Style samples (including stigma) were collected at T_1_, T_2_, and T_5_ stages for RNA-seq analysis. Total RNA was extracted from pooled styles (30 flowers per replicate) using the RNA extraction kit (Mei5bio, Beijing, China), with three biological replicates per stage. RNA concentration and quality were detected using a NanoDrop-2000 ultra-micro spectrophotometer (Thermo Scientific, New York, USA). The prepared RNA samples were sent to Qingdao STD Standard Testing Co., Ltd. (Qingdao, China) for library preparation and sequencing. Stranded mRNA-seq libraries were constructed using poly(A) selection, followed by sequencing on the Illumina NovaSeq 6000 platform with 150 bp paired-end reads. Data filtering was subsequently performed.

### Sequence data analysis

Genome alignment was performed using HISAT2 v2.2.1 with default parameters, indexing the passion fruit reference genome (*Passiflora_edulis*. customer_v1.genome.fa) [24]. Transcript assembly and FPKM-based expression quantification were conducted with StringTie v2.1.4 [25]. Differentially expressed genes (DEGs) were identified using DESeq2 v1.30.1 (CNCB gene IDs) with thresholds of |log_2_FC| ≥ 1 (fold change ≥ 2) and False Discovery Rate (FDR) < 0.05 [26]. The FDR is obtained by correcting for the *p-value*, indicating the significance of the difference. Standardize gene expression levels using FPKM [27]. If FPKM ≥ 1 in at least one sample, it is considered that the gene is expressed. Principal component analysis (PCA) was performed using TBtools v2.327 with default settings [28], including all expressed genes after removing outliers exceeding 3 standard deviations from the mean.

### KEGG analysis

KEGG pathway enrichment analysis of differentially expressed genes was performed using the Kyoto Encyclopedia of Genes and Genomes database [29], with significantly enriched pathways identified at a threshold of adjusted *p*-value < 0.05 (Benjamini-Hochberg correction). Results were visualized using ClusterProfiler v4.0 [30].

### Determination of lignin content

Style samples were obtained from T_1_, T_2_, and T_5_ stages. Cell wall substance was extracted from style samples using the cell wall substances extraction kit (Solarbio Science & Technology Co., Ltd, Beijing, China). After drying and grinding the cell wall material, Pass through a 30–50 mesh sieve, and weigh 5 mg into a 1.5 ml centrifuge tube. Acetylate the screened cell wall material according to the instructions of the lignin content detection kit (Solarbio Science & Technology Co., Ltd, Beijing, China) to obtain the supernatant to be tested. Take 20 µl supernatant add it to 980 µl acetic acid. Mix and use a UV spectrophotometer to measure the absorbance at 280 nm. Finally, calculate the lignin content in the sample according to the formula provided in the manual.

### NBT and DAB staining

NBT and DAB were used to stain and observe the styles of the T_1_, T_2_, and T_5_ stages, respectively. DAB was used to measure the hydrogen peroxide content.

### Determination of total antioxidant capacity

Obtained style samples from the T_1_, T_2_, and T_5_ stages, and then ground them into a homogenous solution using a PBS solution (0.01 M, PH = 7.4). After centrifugation, collected the supernatant. Utilized the BCA protein concentration assay kit (Elabscience Biotechnology Co., Ltd., Wuhan, China) to determine the total protein concentration of the extract. Assessed the total antioxidant capacity of the samples according to the instructions in the T-AOC colorimetric test kit (Elabscience Biotechnology Co., Ltd., Wuhan, China).

### Determination of endogenous ABA and JA content

Obtained style samples from the T_1_, T_2_, and T_5_ stages, determined the endogenous ABA content using liquid chromatography-tandem mass spectrometry, following the procedures modified from those described by Ross et al., [31]. Similarly, the endogenous JA content in the style samples was determined using liquid chromatography-tandem mass spectrometry, based on the modified procedures introduced by Liu et al., [32].

###  qRT-PCR analysis

Extracted total RNA according to Materials and Methods 4.2, First-strand cDNA was synthesized using a Hifair AdvanceFast 1 st Strand cDNA Synthesis Kit (No Dye)(Yeasen Biotechnology (Shanghai) Co., Ltd, Shanghai, China). qRT-PCR was carried out in triplicate for each sample using Hieff qPCR SYBR Green Master Mix (Low Rox Plus (Beijing Zomanbio Biotechnology Co., Ltd, Beijing, China) on an ABI Life Q1 Plus real-time fluorescent quantitative PCR instrument (Applied Biosystems, Waltham, USA). The *ef1* was used as an internal control. The primer sequences used are listed in Table S7.

## Results

### Microscopic structure of passion fruit style and stigma

**Fig. 1:**
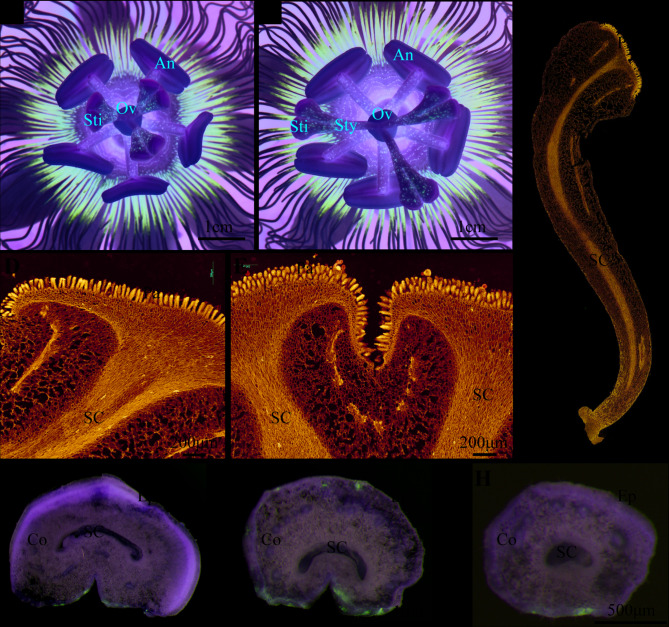
Microstructure of the style and stigma in ‘Tainong 1’. (**A**) and (**B**) are aerial views of ‘Tainong 1’ growing at different times after flowering, Sti, Stigma; Style, Style; Ov, Ovary; An, Anther. **C** Scanning image of paraffin section of the style, Pa, Papilla; Ep, Epidermis; SC, Style Channel, Bar=2mm. **D**-**E** Scanning image of the paraffin section of the stigma, longitudinal section along the abdominal sulcus (**D**) and transverse section (**E**), Bar=200μm. **F**-**H** Cross sections at different positions of the style, (**F**) is the cross section at point a, Bar=1mm, (**G**) is the cross section at point b, Bar=1mm, (**H**) is the cross section at point c, Bar=500μm, Co, Cortex

When the purple passion fruit ‘Tainong 1’ blooms, its pistil has fully developed and consists of three styles. Each style exhibits a fused basal zone connecting to the ovarian apex, a terminal heart-shaped stigma (pollen-receptive surface), and a prominent ventral sulcus (longitudinal groove) that originates from the stigma and gradually diminishes toward the mid-style region (Fig. 1A-B). In order to further observe the structure of the style of ‘Tainong 1’, we performed paraffin sectioning. Our observations revealed that the stigma surface contained densely packed papillary cells (also called mastoid cells; specialized secretory cells displaying elongated morphology). Beneath this layer originated the stylar canal (a pollen tube-guiding tubular structure), which extended through the style to connect with the ovarian locule (Fig. 1C-D). Transverse sections demonstrated that the ventral sulcus forms two stylar channels near the stigma base (Fig. 1E). To verify the number of style channels in the style of ‘Tainong 1’, we performed serial sectioning along the style length (positions a-c in Fig.S1). The results demonstrated a gradual transition from C-shaped channels in the ventral sulcus region to O-shaped configurations in mid-style regions as the sulcal depth decreased. (Fig. 1F-H). At the same time, observation of the transverse section of the style reveals that the style is composed of the outer epidermis, basic tissue, and inner epidermis of the style duct from the outside to the inside (Fig. 1F-H).

### The style exhibits bending movement after flowering

**Fig. 2:**
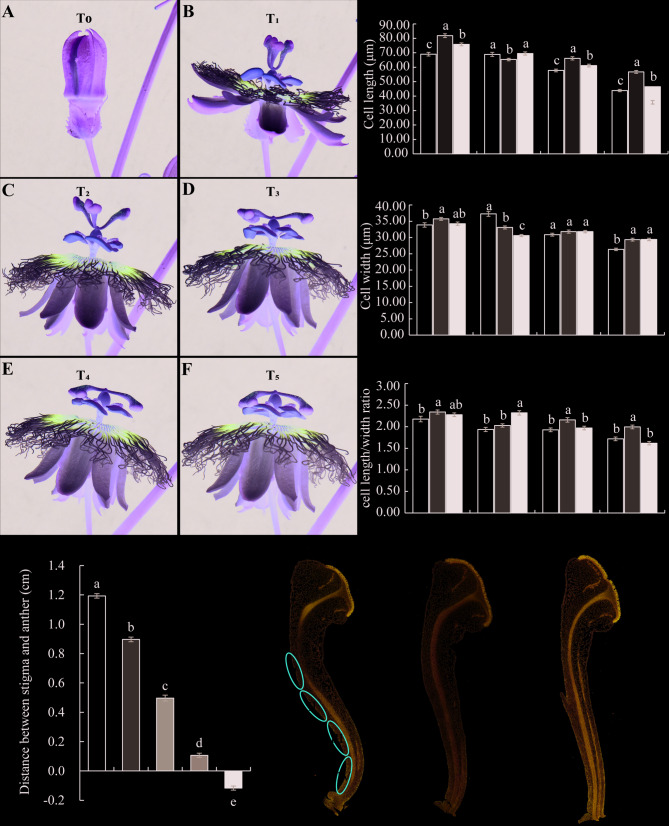
Phenotypic analysis of the bending movement of the style. **A**-**F** Before flowering (T_0_, A), flowering time (T_1_, B), 30 minutes after flowering (T_2_, C), 60 minutes after flowering (T_3_, D), 90 minutes after flowering (T_4_, E), and 120 minutes after flowering (T_5_, F), the shape and spatial position of the style. **G** The distance between the stigma and anthers at T_1_, T_2_, T_3_, T_4_, and T_5_ stages. **H**-**J** Scanned images of paraffin sections of the style at T_1_ (**H**), T_2_ (**I**), and T_5_ (**J**) stages. The red oval shape represents the four parts of the style near the axis (I, upper quarter of the style; II, upper middle part of the style; III, lower middle part of the style; IV, lower quarter of the style), Bar=2mm. (**K**-**M**) The cell length (**K**), cell width (**L**), and cell length/width ratio (**M**) at four parts (I, II, III, and IV) of the style near the axis. Values are shown as means ± SE, different lowercase letters above the column indicating significant differences, columns with the same letter means no significant differences, with different letters means significant differences between groups (*P<0.05*)

In order to explore the mechanism of bending movement of passionflower style, we conducted dynamic observations on the flowering process of ‘Tainong 1’ (T_1_- T_5_) by counting the distance between the stigma and the anther. The results show that the style is in an upright state during the T_1_ stage, and the stigma is the farthest distance from the anther. As the flower bloom time extends, the style shows top-down spatial displacement, especially during the T_5_ stage, the stigma is completely buried in the anther (Fig. 2A-G). In order to observe the factors that cause spatial changes in the style more clearly, we conducted paraffin sectioning of the styles at T_1_, T_2_, and T_5_ stages and found that the style presented an S-shaped at T_1_ stage, which gradually weakened with the development process, and basically became straighter during the T_5_ stage (Fig. 2H-J). Statistical analysis of the cell length, cell width, and aspect ratio in the four proximal parts of the style (I, the upper quarter of the style; II, the upper middle part of the style; III, the lower middle part of the style; IV, the lower quarter of the style) showed significant regional heterogeneity (one-way ANOVA, *p* < 0.05). During petal unfolding, proximal-side cells synchronously underwent longitudinal elongation, lateral expansion, and aspect ratio reduction. These modifications were most pronounced in the lower style quadrant (region IV), where initial cellular dimensions showed significant inter-regional differences (*p* < 0.05 by Student’s t - test; Fig. 2K-M).

### Transcriptome analysis of styles at different stages

**Fig. 3:**
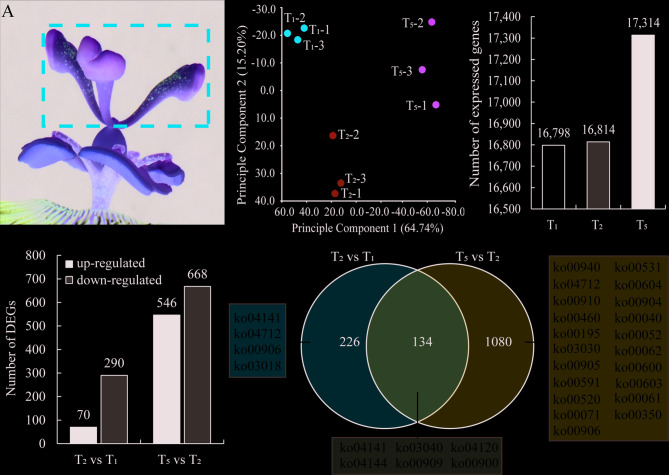
Transcriptome analysis of styles at T_1_, T_2_, and T_5_ stages. **A** Styles (including stigma) sample schematic for RNA extraction. **B** Principal component analysis of the gene expression profiles of styles at T_1_, T_2_, and T_5_ stages. **C** The number of expressed genes (FPKM ≥ 1) identified of the styles at T_1_, T_2_, and T_5_ stages. **D** Comparison of the number of up-regulated and down-regulated DEGs in the T_2_ vs T_1_ and T_5_ vs T_2_ groups. **E** Venn diagram drawn using the DEGs in the T_2_ vs T_1_ and T_5_ vs T_2_ groups, the numbers in different colored box represents the functional pathways enriched by KEGG analysis of DEGs after Venn analysis

In order to further investigate the influencing factors of style bending during the flowering process of passion fruit, we extracted total RNA from style samples at T_1_, T_2_, and T_5_ stages and sequenced (Fig. 3A). The results of principal component analysis showed that the biological repeated clustering of the three different period style samples was good, indicating the reliability of RNA Seq data (Fig. 3B).

A total of 603,427,148 clean reads were obtained by transcriptome sequencing, with each sample having reads ranging from 55,020,322 to 78,547,664, accounting for 84.77% to 86.56% of the original unfiltered total reads. After comparing clean reads to the reference genome of passion fruit (Passiflora_edulis. customer_v1.genome.fa), calculate the FPKM value of 28,612 genes based on the number of reads and fragment length. We consider genes with an FPKM value ≥ 1 as expression genes and find that the number of expression genes in the T_1_ and T_2_ stages is very similar, and their number of expression genes is less than that in the T_5_ stage, with the T_1_ stage having the least expression genes. This also indicates that as the passion fruit style bending, genes in the style are induced to varying degrees of expression, especially during the T_5_ stage when the number of expressed genes is highest (Fig. 3C; Table 1). By comparing the gene expression patterns of styles at different stages through cluster analysis, it was found that there were significant changes in gene expression patterns between styles at different stages (Fig. S2).

**Table 1:** Transcriptome sequencing data statistics

Lines	Rep	Total Reads	Rate of Total Mapped Reads (%)	Num. of Expressed Genes	Rate of Expressed Genes (%)
**T**_**1**_	1	73,684,426	85.44	16,716	58
	2	70,878,430	86.22	16,776	59
	3	68,692,394	85.82	16,901	59
**T**_**2**_	1	78,547,664	86.28	16,847	59
	2	66,705,156	86.1	16,781	59
	3	58,673,328	86.56	16,815	59
**T**_**5**_	1	60,908,196	85.58	17,300	60
	2	55,020,322	84.77	17,195	60
	3	70,317,232	85.3	17,448	61

Total reads: the number of clean reads after filtering the original data; rate of total mapped reads (%): the ratio of the remaining reads after filtering to the original unfiltered reads; num. of expressed genes: total number of expressed genes with FPKM ≥ 1; rate of expressed genes (%): the proportion of the total number of expressed genes with FPKM ≥ 1 to the total number of genes

Using Fold Change ≥ 2 and false discovery rate (FDR) ≤ 0.05 as criteria, DEGs were screened between T_1_ and T_2_ stages (T_2_ vs. T_1_), as well as between T_2_ and T_5_ stages (T_2_ vs. T_1_). 360 DEGs were found in the T_2_ vs. T_1_ group, of which 70 were upregulated and 290 were downregulated. In the T_5_ vs. T_2_ group, there were 1,214 DEGs, of which 546 were upregulated and 668 were downregulated (Fig. 3D). In addition, volcano plots confirmed the distribution pattern of these DEGs across comparison groups (Fig. S3).

In order to investigate the genes and pathways that affect style bending movement in different groups, we performed Venn plot analysis on the DEGs of two groups. The results showed that 226 and 1080 DEGs, respectively, corresponded specifically to the T_2_ vs. T_1_ and T_5_ vs. T_2_ groups (Fig. 3E, Table S2-S3). In addition, we also identified 134 DEGs were co-existing in both comparison groups (T_2_ vs. T_1_ and T_5_ vs. T_2_), which represent candidate genes potentially involved in the entire style bending process (Fig. 3E, Table S1). These shared DEGs were selected solely based on their simultaneous detection in both comparisons.

In order to further investigate the functional pathways of these 134 co-responsive DEGs, we conducted KEGG pathway enrichment analysis and found that they were enriched into 6 pathways, including “Protein processing in endoplasmic reticulum” (ko04141), “Ubiquitin mediated proteolysis” (ko04120), “Endocytosis” (ko04144), and “Sesquiterpenoid and triterpenoid biosynthesis” (ko00909) (Fig. S4A, Table S4). Through searching for gene functional annotations, it was found that 18 DEGs were enriched in the “Protein processing in endoplasmic reticulum” and “Endocytosis” pathways, many of which were heat shock proteins (HSPs), with a higher proportion of HSP70 protein (Table 2). In addition, we also found that three upregulated DEGs were enriched in the Sesquiterpenoid and triterpenoid biosynthesis pathway (Table 2). HSPs are induced to express by temperature stimulation to maintain normal physiological activities of cells. Terpenoids are the largest class of volatile organic compounds in plants and have the function of attracting insects for pollination. This also preliminarily indicates that during the bending movement of the style, the style will be affected by environmental temperature, and it will also induce insects to pollinate it by emitting volatile odors.

**Table 2:** Common DEGs in two groups enriched by the KEGG pathway (partial)

Description	Gene ID	FPKM			T_2_ vs. T_1_		T_5_ vs. T_2_		KEGG Annotation
		T_1_	T_2_	T_5_	FDR	log_2_FC	FDR	log_2_FC	
**Sesquiterpenoid and triterpenoid biosynthesis**	maker-LG06-augustus-gene-30.24	0.408898	1.003999	7.735517	3.91E-05	1.31224	7.76E-21	2.812607	probable terpene synthase 6
	maker-LG06-snap-gene-32.13	0.995293	2.126934	19.80686	0.007789	1.123222	2.63E-38	3.07943	probable terpene synthase 6
	maker-LG08-snap-gene-1093.3	0.652598	1.505193	21.84304	3.00E-06	1.23136	2.41E-53	3.728275	squalene monooxygenase-like
**Ubiquitin mediated proteolysis**	NewGene_5848	12.25933	51.91696	12.25202	7.06E-09	2.117093	5.91E-14	−2.21366	polyubiquitin 4
	maker-LG01-snap-gene-946.8	29.65188	81.63331	20.09136	6.59E-15	1.48485	1.64E-16	−2.15127	probable E3 ubiquitin-protein ligase HERC4 isoform X1, Ultraviolet-B receptor UVR8
	maker-LG05-augustus-gene-1372.12	38.14839	82.62428	8.838019	6.17E-12	1.14164	2.99E-51	−3.35205	probable E3 ubiquitin-protein ligase HERC4 isoform X1, Ultraviolet-B receptor UVR8
	snap_masked-LG01-processed-gene-1786.16	4.671394	10.71011	4.200778	1.25E-09	1.221336	4.90E-08	−1.47453	WD40 repeat protein
	snap_masked-LG03-processed-gene-155.6	185.2508	375.162	141.9134	1.73E-08	1.046148	1.11E-10	−1.53101	ubiquitin-conjugating enzyme E2 28-like
**Protein processing in endoplasmic reticulum**	NewGene_2916	3.337416	52.59632	2.970704	1.56E-27	3.98038	4.64E-23	−4.23303	heat shock protein 83-like
	NewGene_297	352.2693	1067.567	359.2782	3.77E-63	1.626323	4.72E-12	−1.69953	heat shock cognate protein 80
	NewGene_5535	12.88159	309.8564	52.31252	5.64E-37	4.620333	0.001594	−2.73218	HSP20 family protein
**Endocytosis**	augustus_masked-LG05-processed-gene-1369.0	4.358277	84.62514	4.062983	6.96E-25	4.297718	7.43E-08	−4.50812	heat shock 70 kDa protein
	maker-LG01-snap-gene-1736.28	562.8715	1742.47	741.9601	3.42E-22	1.656478	0.005475	−1.36168	heat shock cognate 70 kDa protein 2
	maker-LG05-augustus-gene-1369.32	343.4672	1079.799	247.6856	4.50E-58	1.680147	1.22E-20	−2.25255	heat shock cognate 70 kDa protein 2
	maker-LG07-augustus-gene-1237.60	53.3629	276.738	89.01974	3.82E-67	2.400737	1.71E-12	−1.76351	stromal 70 kDa heat shock-related protein, chloroplastic
	snap_masked-LG04-processed-gene-1211.0	0.182998	5.376168	1.372995	8.11E-39	4.863112	0.003936	−2.09321	heat shock 70 kDa protein-like

### The bending movement of passion fruit styles may be affected by thermal stimulation

T_1_ to T_2_ is the initial stage of style bending movement. Compared with the T_5_ stage, during this stage, there were 226 specifically expressed DEGs, of which 56 were upregulated and 170 were downregulated (Table S2). T_2_ to T_5_ is the stage with the highest degree of style bending movement. During this stage, 1,080 DEGs were specifically expressed, of which 456 were upregulated and 624 were downregulated (Table S3).

**Fig. 4:**
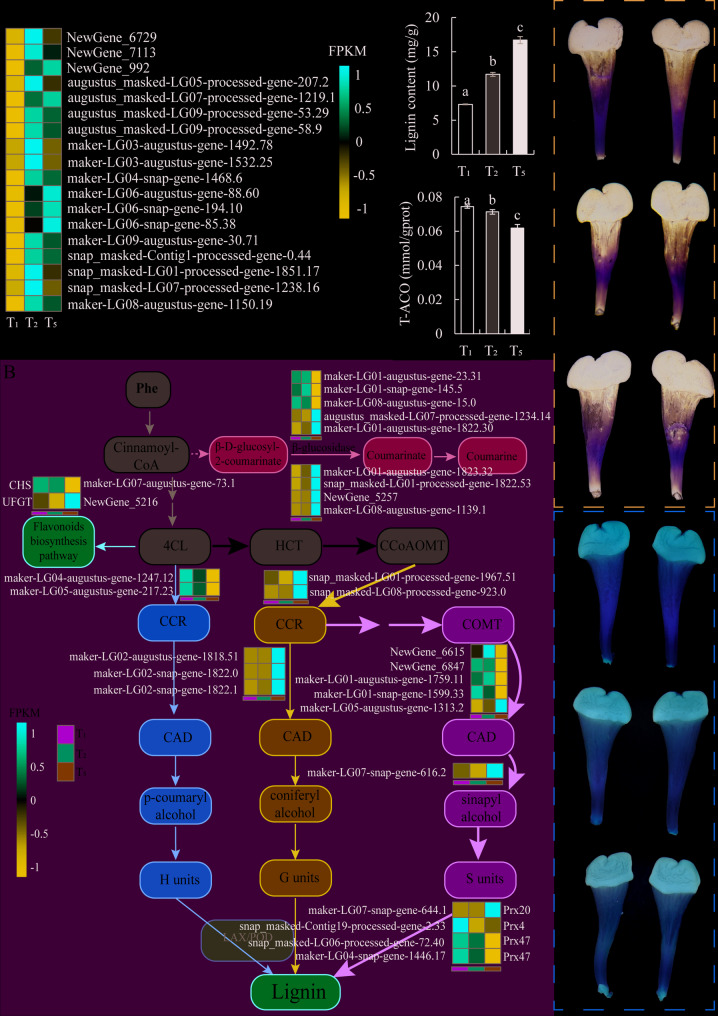
Expression analysis and partial functional validation of specifically expressed DEGs in styles at different stages. **A** Clustering heatmap of specific DEGs enriched with heat shock protein (HSP) function in the T_2_ vs T_1_ group (**B**) Clustering heatmap of specific DEGs enriched in lignin biosynthesis pathways in T_5_ vs T_2_ groups. According to the standardized FPKM, red and blue represent high abundance and low abundance, respectively. Phe, Phenylalanine; 4CL, 4-coumaric acid coenzyme a ligase; HCT, hydroxycinnamoyl transferase; CCoAOMT, caffeoyl-CoA O-methyltransferase; CCR, cinnamoyl CoA reductase; COMT, catechol-O-methyltransferase; CAD, cinnamyl alcohol dehydrogenase; H units, p-hydroxyphenyl lignin; G units, guaiacyl lignin; S units, syringyl lignin; CHS, Chalcone Synthase; UFGT, flavonoid-3-O-glucosyltransferase; LAX, Laccase; POD, Peroxidase. (**C**-**F**) The Lignin content (**C**), the total antioxidant capacity (T-ACO, D), nitrobluetetrazolium (NBT) staining (**E**) and 3, 3-diaminobenzidine (DAB) staining (**F**) in the style at T_1_, T_2_ and T_5_ stages. Values are shown as means ± SE, different lowercase letters above the column indicating significant differences, columns with the same letter means no significant differences, with different letters means significant differences between groups (*P<0.05*)

To further determine the functional pathways of these specifically expressed DEGs at different stages of style bending movement, we performed KEGG pathway enrichment analysis on 1,306 DEGs from these two stages (Fig. S4B-C, Table S5-S6). The analysis results showed that in the T_2_ vs. T_1_ group, specifically expressed genes were mainly enriched in the “Protein processing in endoplasmic reticulum” (ko04141), “Circadian rhythm plant” (ko04712), “Carotenoid biosynthesis” (ko00906), and “RNA degradation” (ko03018) pathways (Fig. S4B, Table S5). Among them, 18 DEGs were enriched in the “Protein processing in endoplasmic reticulum” pathway. Normalized and heat mapped the expression levels of these 18 DEGs in three different style samples. The results showed that compared to the T_1_ stage, all DEGs were induced to be upregulated in the T_2_ stage. Except for *NewGene_992*, *augustus_maked-LG07 processed gene 1219.1*, *maker-LG06 augustus gene 88.60*, *maker-LG06 snap gene 194.10*, and *maker-LG06 snap gene 85.38*, which were continuously upregulated in the T_5_ stage. Other DEGs showed different degrees of downregulation in the T_5_ stage compared to the T_2_ stage (Fig. 4A). Gene functional annotation queries were performed on these 5 DEGs, and it was found that NewGene-992, maker LG06 augustus gene 88.60, and maker LG06 snap gene 85.38 are all HSP20 family proteins. maker LG06 snap gene 194.10 is an HSP90 family protein, and augustus_maked-LG07 processed gene 1219.1 is a DnaJ family protein. HSPs can be induced by heat stimulation to maintain cell growth and development. In this study, as the style bends, HSPs and their molecular chaperones continue to be upregulated. This further suggests that the process of style bending movement may be affected by heat stimulation.

### The bending movement of passion fruit styles may be caused by the accumulation of lignin

At the same time, we analyzed and found that DEGs specifically expressed in the T_5_ vs. T_2_ group were enriched in 21 KEGG pathways, of which 30 DEGs were significantly enriched in the “Phenolpropanoid biosynthesis” (ko00940) pathway, which was the pathway with the most enriched DEGs (Fig. S4C, Table S6). By conducting gene functional annotation queries on these 30 DEGs, it was found that they are involved in the lignin biosynthesis pathway and belong to the 7 types of enzymes in the lignin biosynthesis pathway. These enzymes can gradually catalyze the synthesis of stable lignin from phenylalanine (Fig. 4B). Among them, 4-coumaric acid CoA ligase (4CL) and shikimic acid/quinic acid hydroxycinnamoyl transferase (HCT) can enable coumaric acid CoA to be used for lignin synthesis. Cinnamoyl CoA reductase (CCR), caffeic acid O-methyltransferase (COMT), and cinnamyl alcohol dehydrogenase (CAD) are involved in lignin monomer synthesis. Peroxidase (POD) is involved in the final step of lignin formation. In addition, β- Glucosidase is located in the cell wall of the developing xylem and can affect the formation of lignin. We normalized the expression data of the same gene for style samples at three different stages and plotted a heatmap. The results showed that most DEGs encoding the lignin biosynthesis pathway were gradually upregulated with the bending movement of the style, especially reaching their peak expression level at T_5_ stage (Fig. 4B). To verify whether the accumulation trend of lignin during the bending movement of the style is consistent with transcriptome data, we used UV spectrophotometry to measure the content of lignin in styles at three different stages. The measurement revealed that lignin gradually accumulated in the styles during bending progression, peaking at the T_5_ stage. Specifically, lignin content increased by 60% from T_1_ to T_2_ stage and by 43% from T_2_ to T_5_ stage (Fig. 4C). The accumulation of lignin is beneficial for enhancing the mechanical strength of plants and making them less prone to lodging, which is consistent with the phenotype observed in our paraffin sections that the degree of S-shaped of the style itself weakens with the bending movement of the style (Figs. 2H-J and 4C). Based on the above results, it can be concluded that after the flowering of passion fruit, genes related to the lignin synthesis pathway are induced to be upregulated in the style, leading to the accumulation of lignin in the style and an increase in the mechanical strength of the style. The S-shaped bending degree gradually weakens, resulting in the formation of the style itself and spatial bending movement.

In addition, we found four peroxidase genes, except for *prx20* (*maker LG04 snap gene 1446.17*) which showed a gradually upregulated trend during style bending movement, the other three showed a gradually downregulated trend, especially with the highest expression level at T_1_ stage (Fig. 4B). Meanwhile, we also found that an enriched chalcone synthase (CHS) gene (*maker LG07 augustus gene 73.1*) and two 4CL genes (*maker LG04 augustus gene 1247.12* and *maker LG05 augustus gene 217.23*) also showed a gradually downregulated expression trend (Fig. 4B). 4CL, as an upstream functional enzyme in the phenylalanine metabolism pathway, participates in flavonoid biosynthesis and lignin biosynthesis. Chalcone synthase is a key enzyme in the flavonoid biosynthesis pathway and has strong antioxidant capacity. Therefore, we speculate that prx20 may mainly participate in lignin biosynthesis, while the other three catalases may play an antioxidant role. The downregulation trend of the phenotype of two 4CLs in the bending movement of the style may be due to their role in the flavonoid biosynthesis pathway, rather than in the lignin biosynthesis pathway. In order to further verify whether the three PODs and CHS have antioxidant effects in the style, we performed nitrobluetetrazolium (NBT) and 3,3-diaminobenzidine (DAB) staining and total antioxidant capacity (T-ACO) measurement on three samples of styles at different stages. The results showed that compared to the styles at T_1_ stage, the styles at T_2_ and T_5_ stages showed deeper and larger staining areas, especially the darkest color area at T_5_ stage (Fig. 4E-F). As expected, the T_1_ style exhibited significantly stronger antioxidant capacity compared to the T_2_ and T_5_ stages, while the T_5_ style exhibited the weakest antioxidant capacity (Fig. 4D). The above results also indicate that during the bending movement of the style, there is a decrease in the antioxidant capacity of the style.

### The bending movement of passion fruit styles may be mediated by endogenous hormones

**Fig. 5:**
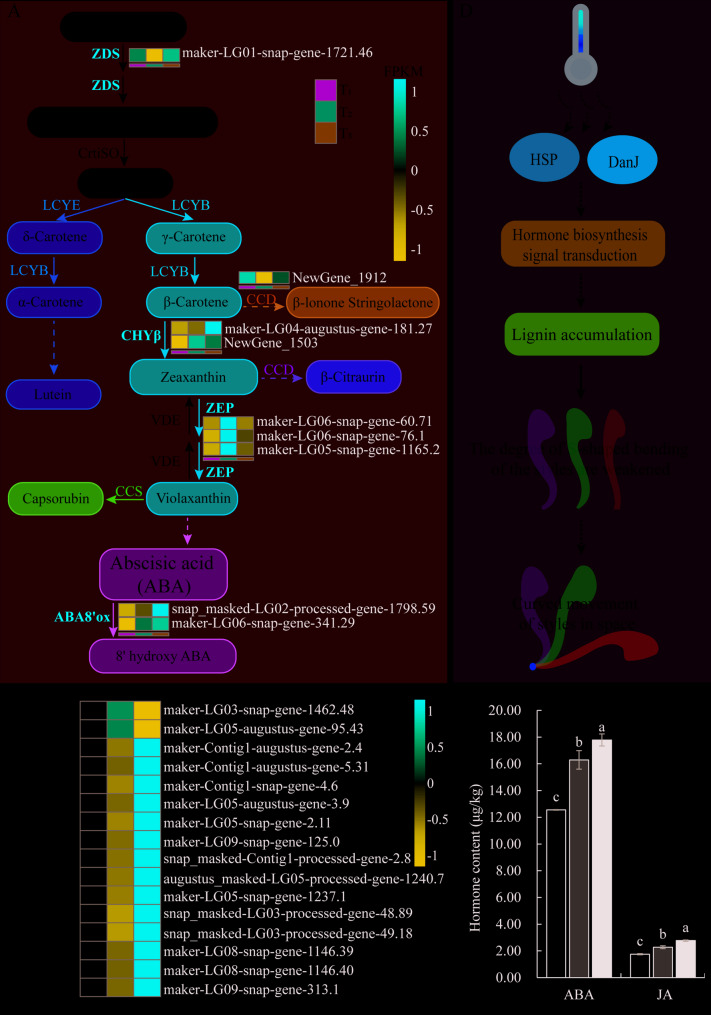
Hormone biosynthesis metabolic pathways and style bending movement patterns. **A** Clustering heatmap of specific DEGs enriched in the abscisic acid (ABA) biosynthetic metabolic pathway in T_2_ vs T_1_ and T_5_ vs T_2_ groups, respectively. ZDS, ζ-carotene desaturase; CrtiSO, Carotenoid isomerase; LCYE, lycopene ε-cyclase; LCYB, Lycopene β-cyclase; CCD, carotenoid cleavage dixoygenases; CHYβ, carotene β-hydroxylase; VDE, violaxanthin de-epoxidase; ZEP, Zeaxanthin epoxidase; CCS, Capsanthin/Capsorubin synthase; ABA8’ox, 8'.ABA hydroxylase. **B** Clustering heatmap of specific DEGs enriched in brassinosteroids (BRs), jasmonic acid (JA), and gibberellin (GA) biosynthetic metabolic pathways in T_5_ vs T_2_ groups. According to the standardized FPKM, red and blue represent high abundance and low abundance, respectively. **C** The ABA and JA content in the style at T_1_, T_2_ and T_5_ stages. Values are shown as means± SE, different lowercase letters above the column indicating significant differences, columns with the same letter means no significant differences, with different letters means significant differences between groups (*P<0.05*).**D** Schematic diagram illustrating the regulation of style bending movement by the temperature-hormone-lignin module

Further analysis of the KEGG pathway enrichment results of specific DEGs from T_1_ to T_2_ and T_2_ to T_5_ stages revealed that in both stages, DEGs were enriched in the “carotenoid biosynthesis” (ko00906) pathway (Fig. S4B-C, Table S5-S6). The “carotenoid biosynthesis pathway” is the biosynthetic metabolic pathway of abscisic acid (ABA). Carotenoids are precursors of ABA, which is the substrate of 8’ ABA hydroxylase (ABA8’ox). Both Carotene β-Hydroxylase (CHY-β) and Zeaxanthin epoxidase (ZEP) are functional enzymes involved in the ABA biosynthesis pathway, and upregulation of expression promotes an increase in endogenous ABA content in the style. Both ABA8’ox were upregulated with the increase of ABA content, especially at the T_5_ stage, with the highest expression level (Fig. 5A). Therefore, we speculate that the accumulation of endogenous ABA is also accompanied by the bending process of the style. To verify this hypothesis, we measured the content of endogenous ABA in three different stages styles. The results revealed a biphasic accumulation pattern: a sharp 30% increase from T_1_ to T_2_ stage, followed by a gradual 9% rise from T_2_ to T_5_ stage (Fig. 5C). The above results indicate that ABA biosynthesis related genes are induced to be upregulated during the bending movement of the style, and ABA gradually accumulates, promoting the upregulation of endogenous ABA8’ox expression and participating in the decomposition of endogenous ABA in the style.

In addition, we also found 9 DEGs enriched in “Brassinosteroid biosynthesis” (ko00905) and 5 DEGs enriched in “Linoleic acid metabolism” (ko00591) in the T_5_ vs. T_2_ groups, including 4 DEGs (*13-Lox*, *augustus_mask LG05 processed gene 1240.7* and *maker LG05 snap gene 1237.1*; 9-Lox, *snap_mask LG03 processed gene 48.89* and *snap_mask - processed gene 49.18*) is involved in the biosynthesis of jasmonic acid. 7 DEGs are enriched in “Dieterpenoid biosynthesis” (ko00904), among them, 3 DEGs (*GA2ox*, *maker-LG09-snap-gene-313.1*, *maker-LG04-augustus-gene-181.27* and *snap_masked-LG02-processed-gene-1798.59*) are involved in the conversion of GA1 to GA8 (Fig. S4C, Table S6). Normalize the expression levels of the 16 hormone related genes mentioned above in style samples at three different stages and draw heatmaps. It was found that except for two genes in the “brassinosteroid biosynthesis” pathway that were downregulated with style bending, the other 14 genes were induced to be upregulated, especially in the T_5_ stage, with the highest expression levels (Fig. 5B). Meanwhile, we measured the content of jasmonic acid in style samples at three different stages and found that a 30% increase from T_1_ to T_2_ stage, followed by a further 22% increase from T_2_ to T_5_ stage, with this accumulation trend being consistent with the transcriptome data (Fig. 5C).

### qRT-PCR analysis of DEGs

**Fig. 6:**
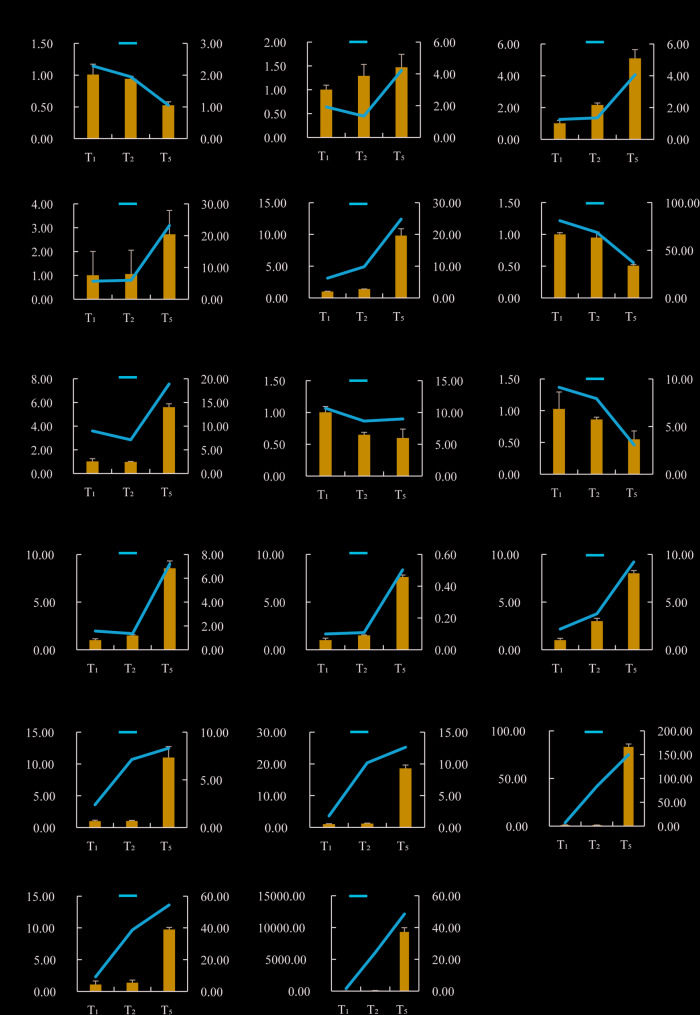
Use qPCR to verify DEGs in the transcriptome of the style at different stages. **A**-**G** is the lignin biosynthesis pathway genes, **H**-**I** is the catalase functional genes, (**J**-**K**) is the circadian rhythm pathway genes, (**L**-**M**) is the abscisic acid synthesis metabolism pathway genes, and (**N**-**Q**) is the heat shock protein functional genes. The column represents the qPCR expression level of genes, and the orange line represents the FPKM of genes transcriptome standardization. *ef1* was used as an internal control, Values are shown as means ± SE, *n*=3

To further validate the accuracy of RNA-seq data, we selected 17 DEGs involved in pathways such as heat stress, lignin accumulation, antioxidant, and ABA decomposition, and used qRT-PCR to detect their expression levels in three different stages of style samples (Table S7). Analysis of qRT-PCR results revealed that the upregulation or downregulation trends of each DEG in the styles at three different stages were consistent with the results of RNA-seq analysis (Fig. 6). This also indicated that the transcriptome data results are accurate and reliable.

## Discussion

### Inherent bending of the style affects its spatial bending movement

Currently, many studies have confirmed that for plants like passion fruit with protruding stigma, the bending movement of the style is beneficial for the combination of stigma and pollen, and plays an important role in the reproductive process [33, 34]. Some researchers believe that the bending movement of passion fruit styles is initiated by a certain substance, which is produced during the development of female gametophytes [35]. However, there is little research on what the substance is and how it is bent through its mechanism. This study conducted dynamic observation and paraffin section observation on the style of purple passion fruit ‘Tainong 1’, and found that while the style was bent in a top-down spatial orientation, the degree of its inherent S-shaped bending was weakened (Fig. 2). From a phenotypic perspective, the spatial bending of the style may primarily result from changes in its inherent bending.

### Lignin accumulation weakens the inherent S-shaped bending of the style

Lignin is one of the important products of plant phenylpropanoid metabolism and a major component of plant cell walls [36]. Lignin accumulates in the stems of crops such as corn, wheat, and rice, which helps to improve their mechanical strength and enhance their lodging resistance [37–40]. In this study, we found through paraffin sectioning that as the style bends, the degree of bending of the style itself weakens (Fig. 2H-J). Meanwhile, transcriptome data of style samples at different stages also showed that lignin biosynthesis related genes were induced to be upregulated during the bending movement of the style (Fig. 4B). The measurement results of lignin content in the styles at different stages are also consistent with the paraffin sections and transcriptome data (Fig. 4C). Current data suggest that the attenuation of the inherent S-shaped bending in styles may be associated with increased lignin accumulation, which typically enhances mechanical rigidity. Furthermore, the lignin-induced reduction in this bending may also contribute to the observed top-down spatial bending of the style. Although these two morphological modifications may be systematically related, current evidence remains inconclusive, warranting further investigation to elucidate their potential mechanistic linkage.

### Temperature may serve as a potential environmental modulator of style bending movement

Flowering is an important physiological phenomenon in the growth and reproduction process of plants, and is a symbol of the transition from vegetative growth to reproductive growth [41]. Plant flowering is regulated by various endogenous and exogenous factors, among which temperature is an important environmental factor affecting plant flowering [42–45]. HSPs are induced to synthesize by heat stimulation, and members of the DanJ family are typical molecular chaperones of HSPs [46]. Pollen, stigma, and style are important components of the stamen and pistil in flower structure. Many studies have shown that temperature has a significant impact on pollen vitality and stigma receptivity [47–50]. High temperature environment can reduce the S-RNase activity of sakura styles, enhance the growth of pollen tubes, and overcome their self-incompatibility [51]. Meanwhile, temperature changes are also a key factor in regulating style elongation [52]. In addition, Yang et al., also found that when the average temperature was greater than 18 ℃ and the average humidity was less than 90%, the bending movement of *Amomum tsaoko*’s style significantly lagged with increasing temperature and decreasing humidity [53]. In this study, we found that during the initial stage of passion fruit style bending movement, a large number of DEGs of the HSP family and DnaJ protein family were upregulated, among which 5 DEGs were continuously upregulated in the T_5_ stage of the style (Fig. 4A). Based on the current research findings, we hypothesize that elevated ambient temperature may upregulate the expression of HSP family genes, thereby potentially regulating the bending movement of styles. However, the precise molecular mechanisms underlying the temperature-HSP-style bending regulatory pathway remain to be elucidated, necessitating further experimental validation of their functional relationship. *Amomum tsaoko* plants prefer shade at an optimal temperature of 15 to 20 ℃, while passion fruit tropical fruits have an optimal growth temperature of 20 to 30 ℃. Due to the differences in temperature adaptability of their plants, *Ammum tsaoko* and passion fruit styles exhibit different bending trends under high temperature environmental stimuli.

### Hormones affect the bending movement of the style

Plant hormones are endogenous metabolites of plants themselves and important signaling factors that run through the entire process of plant growth and development [54, 55]. In the study of the bending movement of passion fruit styles, some researchers have suggested that both IAA and NPA can increase the bending movement speed of the male and female pistil styles of passion fruit *P. sanguinolenta*, and it is believed that the uneven distribution of auxin leads to the bending of the male and female pistil styles of passion fruit *P. mucronate* [56, 57]. ABA as a plant hormone that inhibits growth, is a key factor in balancing endogenous hormones and regulating growth metabolism in plants, exhibiting antagonistic effects against GA [58]. GA is involved in regulating cell growth, and studies have shown that an increase in GA content enhances the lignin content in celery leaves [59], promoting the accumulation of lignin content in winter wheat straw and enhancing its ability to resist lodging. JA as a phytohormone regulating defense and development, enhances lignin accumulation through multiple mechanisms - directly increasing lignin content in jasmine fruits [60] and upregulating HCT-mediated biosynthesis in tea leaves [61]. BRs are growth promoting steroid hormones [62], but it is still unknown whether BRs can promote lignin accumulation.

This study found that the transcriptome data of style bending movement was not enriched in the synthesis and metabolism pathways related to auxin, but rather enriched in DEGs related to the biosynthesis and metabolism pathways of ABA, JA, GA, and BRs (Fig. S4B-C, Table S5-S6). The DEGs related to the ABA synthesis pathway were significantly upregulated in T_2_ stage, but their expression levels showed a downregulation trend in T_5_ stage. At the same time, two ABA8’ox (ABA degrading enzymes) functional genes showed high expression levels in T_5_ stage, inhibiting the accumulation rate of ABA (Fig. 5A). Except for ABA, the other three hormones showed an induced upregulation trend with the bending movement of the style, especially during the T_5_ stage (Fig. 5B).

Plant hormones also play an important role in regulating environmental factor responses [63, 64]. There have been research reports that JA can participate in regulating the response of wheat to high temperature stress [65], exogenous application of GA can alleviate wheat heat stress [66], and exogenous BRs can improve rice pollen vitality under heat stress environment [67]. In addition, Wei et al., found that the interaction between cassava heat shock protein HSP90.9 and WRKY20 and SRS1 can regulate the antagonistic effect between salicylic acid and auxin [68]. Exogenous application of ABA can induce the expression of HSP70 in cucumber, thereby improving plant heat tolerance [69]. However, the signal transduction and regulatory mechanism between HSPs and hormones to promote the accumulation of lignin in the style, leading to the bending of passion fruit style, remains an unknown issue. In the future, we will further explore the effects of different environmental temperatures and exogenous hormones on the bending of passion fruit styles.

Based on the results obtained in this study, combined with the previous research progress mentioned above, we propose a working hypothesis that requires further experimental validation: After the flowering of passion fruit, the style is stimulated by environmental heat factors, which may activate the upregulation of HSPs and their chaperones in the style, enabling endogenous signaling pathways such as ABA, GA, JA, and BRs to play a role in the style. Under the mediation of endogenous hormones, genes related to lignin biosynthesis pathways are induced to be upregulated in the style, potentially leading to lignin accumulation and a reduction in the S-shaped bending degree of the style itself, ultimately forming a spatial bending movement of the style from top to bottom (Fig. 5D). This putative ‘heat-HSP-hormone-lignin’ axis requires validation through: Phenotype and physiological analyses to correlate endogenous hormone levels, lignin accumulation, and bending kinematics under varying temperature and hormone conditions; Molecular investigations to elucidate HSP-hormone interactions and their regulatory effects on lignin biosynthesis pathways; Mechanistic studies to clarify how temperature-hormone-lignin modules coordinate style bending movement.

## Conclusion

Passion fruit faces significant yield limitations due to its approach herkogamy. This study reveals that the dynamic bending movement of the style in ‘Tainong 1’ plays a critical role in optimizing pollination efficiency. Through transcriptomic and physiological analyses, we identified the key regulatory phases governing this process: Early-stage response (T_1_–T_2_): Heat stress triggers the upregulation of heat shock proteins (HSPs) and DnaJ family proteins, suggesting that environmental thermal cues initiate style movement. Late-stage development (T_2_–T_5_): Lignin biosynthesis dominates, with transcriptomic and physiological data confirming that lignin accumulation coincides with the weakening of the style’s S-shaped, likely enhancing mechanical rigidity. Additionally, hormonal regulation—particularly involving abscisic acid, brassinosteroids, jasmonic acid, and gibberellin—further modulates this process, as evidenced by differential gene expression and endogenous hormone measurements. These findings propose a molecular framework wherein thermal stimuli, hormonal signaling, and lignin deposition collectively coordinate style bending to mitigate herkogamy. While this study provides foundational insights, further functional validation is needed to dissect the precise regulatory cascades. Our work highlights potential targets for molecular breeding or cultivation strategies aimed at improving passion fruit yield by optimizing floral architecture. 

## Supplementary Information

Supplementary Material 1

Supplementary Material 2

## Data Availability

The raw sequence data reported in this paper have been deposited in the Genome Sequence Archive (Genomics, Proteomics & Bioinformatics 2021) in National Genomics Data Center (Nucleic Acids Res 2022), China National Center for Bioinformation/Beijing Institute of Genomics, Chinese Academy of Sciences (GSA: CRA016406) that are publicly accessible at https://ngdc.cncb.ac.cn/gsa.
