# Supplementary material for: Transcriptome analysis revealed the potential molecular mechanism of style bending movement in passion fruit (Passiflora Edulis Sims)
Source: BMC Plant Biol. 2025 Oct 29;25:1468. doi: 10.1186/s12870-025-07431-8 (PMC12574184; doi:10.1186/s12870-025-07431-8)
Supplement: Supplementary file 1 — Supplementary Material 1 [file 12870_2025_7431_MOESM1_ESM.pdf]

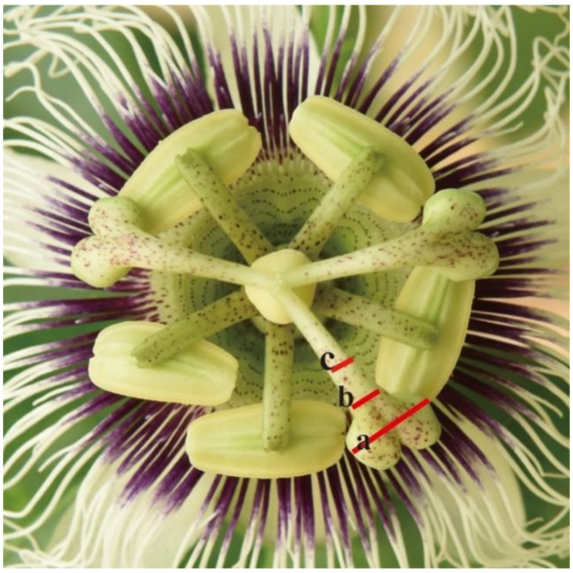

Fig. S1. Aerial views of 'Tainong 1' after flowering. The three red lines corresponding to a, b, and c indicate the three transverse cutting sites of the style from top to bottom.

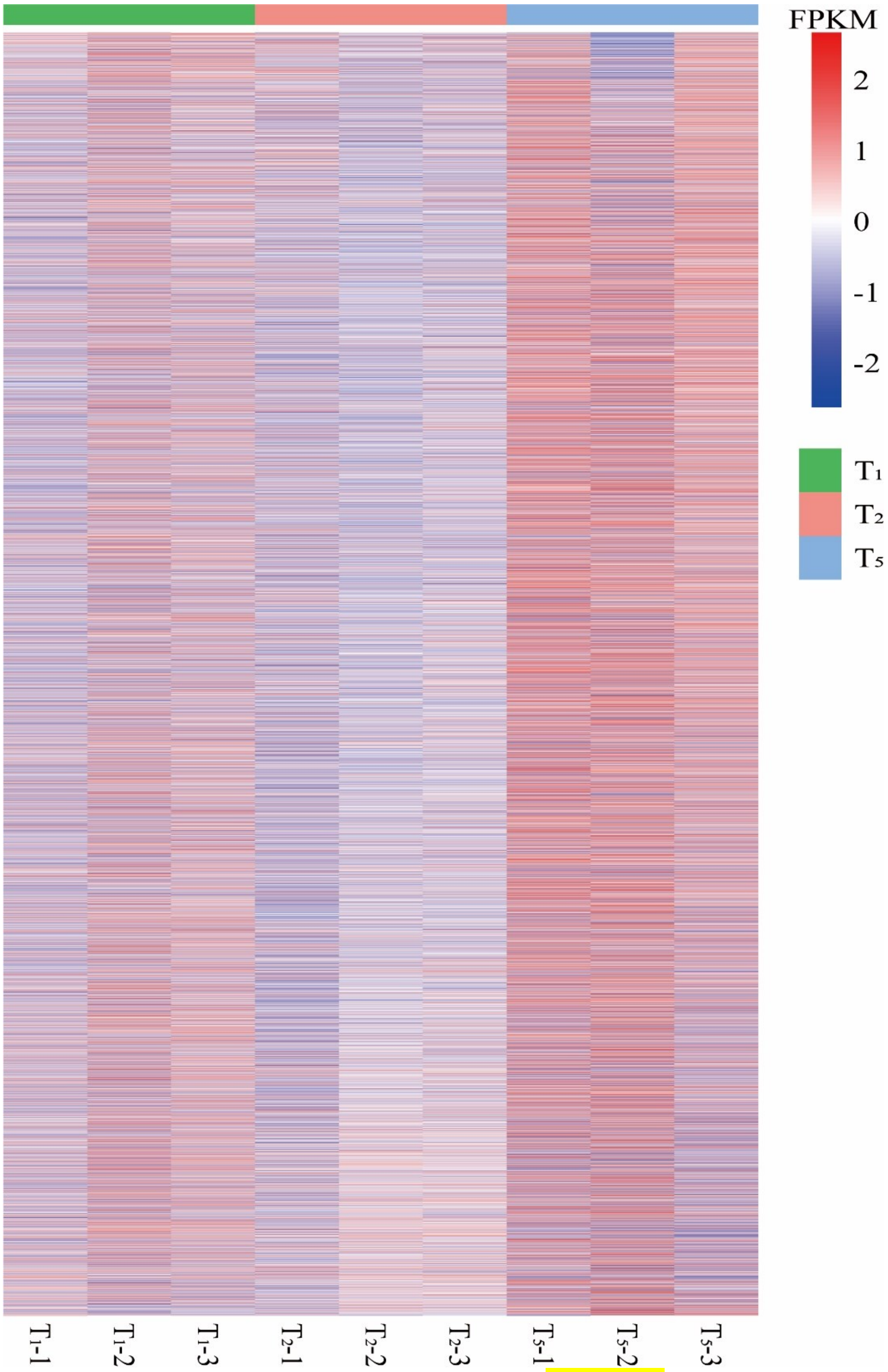

Fig. S2. Heatmap cluster of expressed genes of the style at T_1, T_2 and T_5 stages. Each sample has three compartments, which are three biological replicates. According to the standardized FPKM, red and blue indicate high and low abundance, respectively.

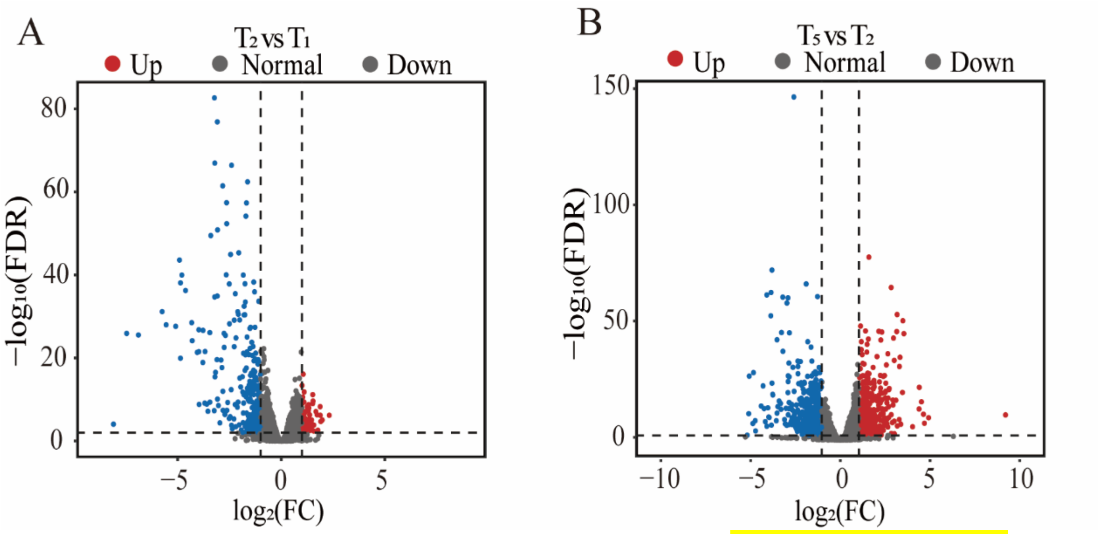

Fig. S3. The volcano plots of differentially expressed genes (DEGs) in T_2 vs T_1 (A) and T_5 vs T_2 (B) groups.

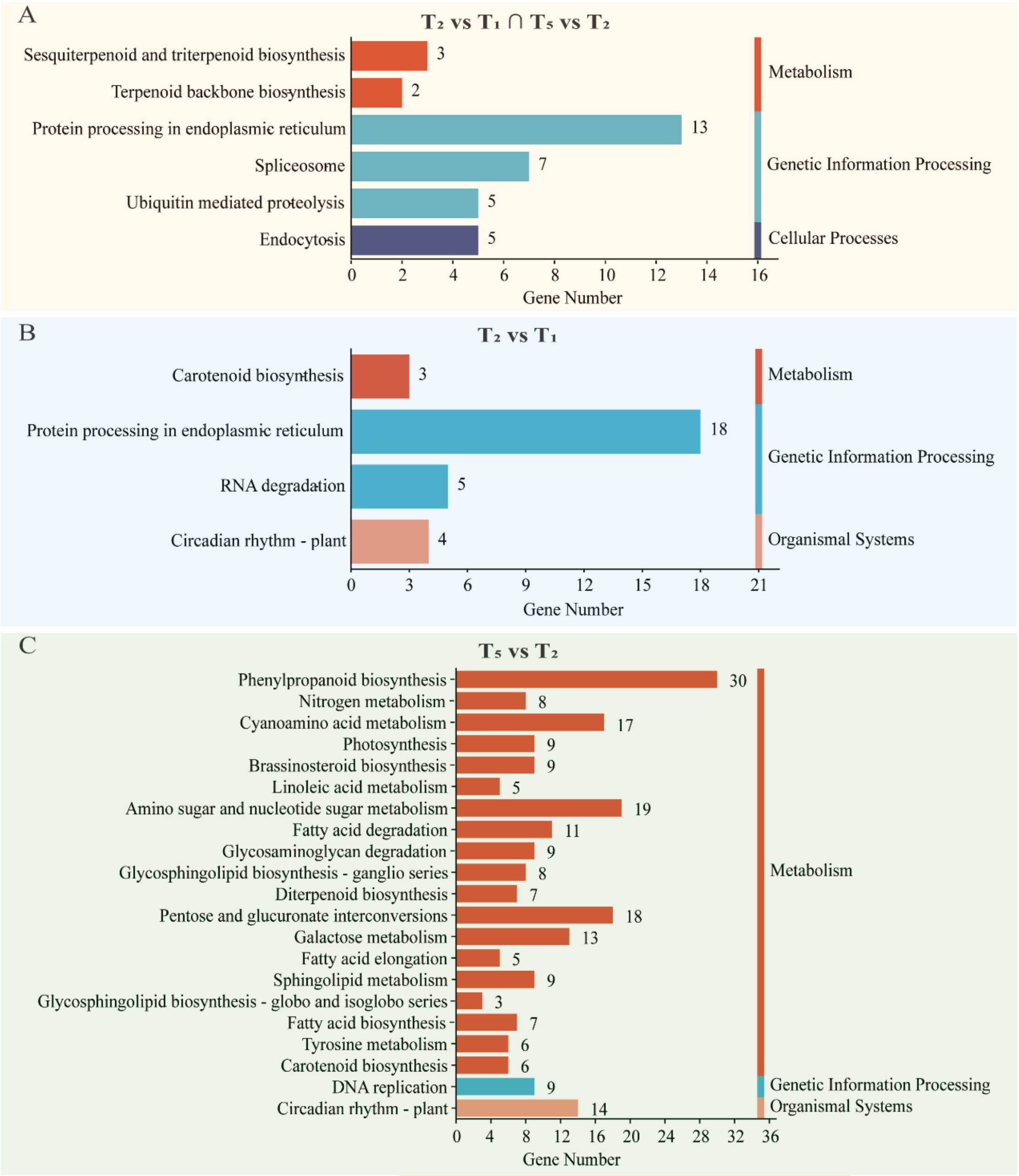

Fig. S4. KEGG analysis of DEGs in T_2 vs T_1 ∩ T_5 vs T_2 (A), T_2 vs T_1 (B) and T_5 vs T_2 (C) groups.
